# siRNA Has Greatly Elevated Mismatch Tolerance at 3′-UTR Sites

**DOI:** 10.1371/journal.pone.0049309

**Published:** 2012-11-08

**Authors:** Na Wei, Lei Zhang, Huang Huang, Yue Chen, Jie Zheng, Xiao Zhou, Fan Yi, Quan Du, Zicai Liang

**Affiliations:** 1 Institute of Molecular Medicine, Peking University, Beijing, China; 2 The State Key Laboratory of Natural and Biomimetic Drugs, School of Pharmaceutical Sciences, Peking University, Beijing, China; University of Florida, United States of America

## Abstract

It has been noted that target sites located in the coding region or the 3′-untranslated region (3′-UTR) can be silenced to significantly different levels by the same siRNA, but little is known about at what specificity the silencing was achieved. In an exploration of positional effects on siRNA specificity by luciferase reporter system, we surprisingly discovered that siRNA had greatly elevated tolerance towards mismatches in target sites in the 3′-UTR of the mRNA compared with the same target sites cloned in the coding region. Assessment of changes in protein and mRNA levels suggested that the differential mismatch tolerance might have resulted from location-specific translational repression in the 3′-UTR. Ablation of argonaute proteins by AGO-specific siRNAs revealed that the AGO2 had major impact on siRNA silencing activity against sites in both coding region and 3′-UTR, while the silencing of nonnucleolytic AGO proteins (AGO1, AGO3 and AGO4) did not significantly affect silencing of sites in either region. This paper revealed the discovery that the specificity of an siRNA can be affected by the location of its target site.

## Introduction

RNA interference (RNAi) is the process of post-transcriptional gene silencing mediated by double-stranded RNA [1]–[3]. Small interfering RNAs (siRNA), a class of 19–21-bp double-stranded RNAs can be incorporated into an RNA-induced silencing complex (RISC) [4] to regulate the expression of target genes by either cleavage of target mRNAs or repressing translation and/or promoting mRNA decay based on the complementarity between the siRNA and its target [5]–[7]. The key components of the RISC are argonaute proteins. In mammalian cells, there are four argonaute proteins (AGO1–AGO4) that are closely related and co-expressed in many tissues and cell types [8]. Structural and biochemical studies indicated that all human AGO proteins are capable of associating with both siRNAs and miRNAs indiscriminately of their sequences and structures [9]–[12]. This is somewhat different from sorting of small RNAs in flies and worms [13]–[15]. Fully complementary siRNAs are loaded into AGO2 in *D. melanogaster* or RDE-1 in *C. elegans*, and these two proteins are specialized for cleavage of perfectly matched targets. However, bulged miRNAs are sorted into AGO1 in files or ALG-1 in worms to function in miRNA pathway. Among human AGO proteins, only AGO2-containing ribonucleoproteins can guide the cleavage of complementary target mRNAs at the site opposite the 10–11th positions of the siRNA guide strand [9], [16], [17]. Recently, all AGOs were proposed to exert translational repression when tethered to an mRNA [18], [19].

RNAi has revolutionized many aspects of life science research as a powerful tool, but the specificity issue has been a matter of concern ever since the early days when siRNA was explored as a potential therapeutic reagent [20]–[22]. Wide-spread mismatch tolerance of siRNA occurs on sites that have mismatches at 1, 2 or 3 nucleotides along the 19-nt region of the siRNA [23]–[26]. Rules of such off-target effects provide us the design guidelines for allele-specific siRNA that can selectively silence certain SNPs causing disease without inhibiting the expression of corresponding wild-type alleles. Another matter of concern is the potency of siRNA, which can be affected by the differential integration of both strands of an siRNA duplex into the RISC [27], by the local structure of the target site [28] and by the siRNA target site locations within an mRNA [19], [29], [30]. Two reports have suggested that siRNAs targeting the 3′-UTR of transcripts could have higher potency than siRNAs interacting with sites located in the coding region [19], [29]. However, the specificity of the siRNAs in such cases was never fully assessed. Previously we have analyzed the pattern of single nucleotide mismatch discrimination in the coding region for 20 siRNA, among which siR-04 and siR-40 was included, on 240 mismatched targeting sites [24], [26]. Therefore, in this study, we chose the included siR-04 and siR-40 as example to compare the function of siRNAs on identical target sites purposely engineered in the coding region and the 3′-UTR of the reporter genes, and quantified the impact of positioning on the potency and especially the specificity of the siRNAs. Two additional new siRNAs siR-26 and siR-206 (which has a guide strand identical to mature sequence of miR-206) were analyzed for verification. We found that specificity of an siRNA can be affected by the location of its target site and the siRNA has greatly elevated mismatch tolerance at 3′-UTR sites.

## Results

### Mismatch Tolerance of siRNA had Greatly Elevated in the 3′-UTR than Coding Region

In our previous studies [24], [26], we have systematically investigated the silencing efficacy of siRNAs on single-nucleotide mismatched targets located in the coding region of a reporter construct. These studies revealed a position-specific discrimination profile across the whole 19-nt target site, and in particular identified the most tolerant and disruptive mismatch at each target position.

**Figure 1:**
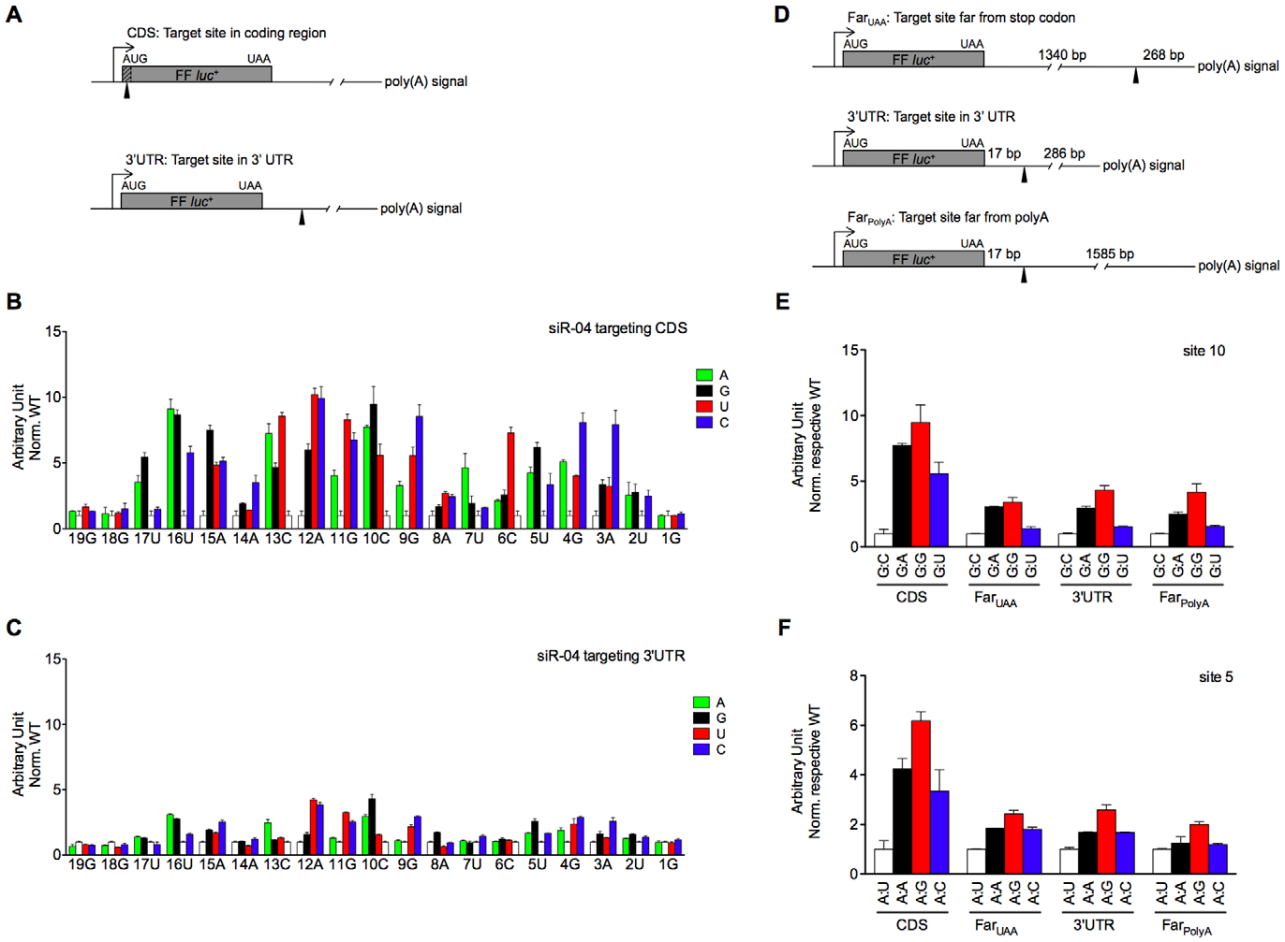
siRNA had greatly elevated mismatch tolerance towards 3′-UTR sites. (A) Schematic diagram of the fusion *firefly* luciferase reporter, with target site inserted in-frame in coding region (CDS) or 3′-UTR, respectively. In the diagram of CDS construct, shown to the left was SV40 promoter and the inserted in-frame AUG start codon, followed by a solid triangle representing the target site and the luciferase gene with authentic UAA stop codon. In the diagram of 3′-UTR construct, original cloning site was destroyed and grafted downstream of the stop codon. (B,C) The normalized silencing efficacies of siR-04 on single-nucleotide mismatched targets in CDS (B) or 3′-UTR (C). The figure was plotted against the position (numbered from the start of the siRNA antisense strand) and the identity of the mismatched nucleotides. The position and the wild-type target sequence were given under the x-axis. The y-axis represented the relative silencing efficacies of siR-04 towards mismatches (colored bars), which were normalized to siRNA towards the perfectly matched site (WT, white bars) to gain the same starting point. The lower of the bars indicated siRNA had stronger silencing activities. (D) Schematic diagram of the fusion *firefly* luciferase reporters, with siRNA target site inserted at different positions relative to the stop codon and poly(A) signal in 3′-UTR. The distances between the target site and the stop codon/poly(A) signal were indicated. (E,F) The normalized silencing efficacies were measured with different vectors harboring siRNA target sites in CDS or at different locations within the 3′-UTR. The target site location and siRNA:mRNA match pattern were given under the x-axis. The y-axis was similar with B and C, which was normalized to each WT construct at either location. Two sites were picked up, site 10 in (E) and site 5 in (F). Error bars represented SD. Data were average values of assays in triplicates, and all experiments were repeated at least twice.

In this study, in order to compare the silencing specificity of siRNA on single-nucleotide mismatched target sites in the coding region versus 3′-UTR, we chose a highly potent siRNA (siR-04) and cloned the corresponding perfectly-matched target site and all possible 57 single-nucleotide mismatched target sites into the coding region or the 3′-UTR of firefly luciferase reporter gene in a eukaryotic expression plasmid (Figure 1A). The absolute activity of the two sets of constructs bearing the target sites in the coding region or the 3′-UTR was equal in the absence of an siRNA (data not shown), and mFold analysis was shown that there were no significant differences in accessibility to the RNAi machinery between the 3′-UTR site and the site located in the coding region of a transcript (Supplementary Figure S1A and B). This was confirmed by the fact that siR-04 had very similar intrinsic activity on perfectly-matched sites located in both regions (see white bars in Supplementary Figure S2A and B). To achieve the same starting point, the silencing efficacy of siR-04 towards mismatches was normalized to that towards the perfectly-matched site. Comparison of the mismatch tolerance of siR-04 on target sites in the coding region versus 3′-UTR, a similar position-specific discrimination profile across the whole 19-nt target site was exhibited. For example, siR-04 was most sensitive to mismatches in the central region of target site, regardless of the target position in the coding region or the 3′-UTR. However, siR-04 showed a greatly elevated mismatch tolerance against sites located in the 3′-UTR, and at the same time the siRNA suffered from astonishing losses of specificity in terms of poor allele discrimination (Figure 1B and C). Still focused on mismatches in the central site, which interrupted the silencing activity of siR-04 on sites located in the coding region of the transcripts, these mismatches were well tolerated when the sites were placed in the 3′-UTR, and silencing activity was only slightly reduced (Supplementary Figure S2A and B).

In order to rule out the influence of siRNA size and sequence, we screened three additional siRNAs (siR-26, siR-40 and siR-206) under the same scrutiny. In total, 366 fusion constructs were prepared and tested. Although different siRNAs had the different extent of elevated mismatch tolerance at 3′-UTR sites compared to the coding region sites, the trend of compromised gene silencing at the 3′-UTR sites was consistent (Supplementary Figure S2C–H). The results demonstrated that elevated mismatch tolerance in siRNA-mediated silencing of 3′-UTR sites is a general phenomenon.

To investigate how the position of the target site relative to the stop codon and polyadenylation signal affects the specificity of siRNA, we inserted mismatched siRNA target sites at different locations in the 3′-UTR. We did this by inserting an irrelevant ∼1300-bp fragment either between the target site and the stop codon, or between the site and the polyadenylation signal (Figure 1D), labeled as Far_UAA_ or Far_polyA_, respectively, then predicated the secondary structure of the three different transcripts (Supplementary Figure S1B–D). It was found that no matter where the sites were placed and what mismatches were involved, elevated mismatch tolerance in the 3′-UTR was independent of siRNA target site location within the 3′-UTR of the transcript (Figure 1E and F).

The lines of evidence demonstrated that elevated mismatch tolerance in siRNA-mediated silencing of 3′-UTR sites is a general phenomenon, which is independent of siRNA target site location within the 3′-UTR.

### Elevated Mismatch Tolerance in the 3′-UTR was Independent of siRNA Concentration

**Figure 2:**
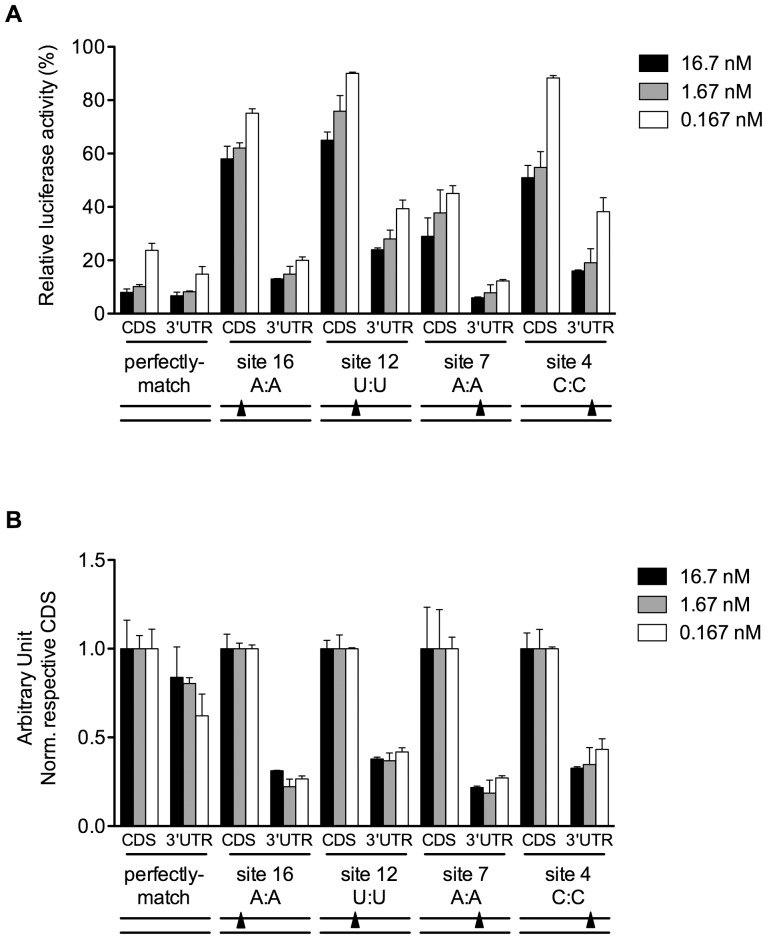
Elevated mismatch tolerance in 3′-UTR was independent of siRNA concentration. Silencing efficacies of siR-04 on matched and single-nucleotide mismatched targets in CDS versus 3′-UTR at the indicated positions (4C, 7A,12U, 16A) were shown as (A) original data and (B) normalized data, by using varying siRNA concentrations from 0.167 to 16.7 nM. The normalized data were calculated by normalizing each original silencing efficacy of siRNA towards 3′-UTR site to that towards CDS site. The target site location and siRNA:mRNA match pattern were given under the x-axis. The y-axis represented siRNA silencing activities. The lower bars indicated siRNA had stronger silencing activities. Below the diagram was a simple graph, double lines indicated siRNA(down):mRNA(up) duplex, the solid triangle indicated the position of mismatch within the target site. Error bars represented SD. Data were average values of assays in triplicates, and all experiments were repeated at least twice.

In order to better compare the impact of mismatches on the efficacy of siRNA on sites located in the coding region versus the 3′-UTR, we made a serial dilution of siR-04 at final concentration of 16.7 nM to 0.167 nM so that any chance of concentration-related bias would be revealed. For the perfectly-matched sites, the silencing activity of siR-04 on the 3′-UTR sites closely tracked that on sites in the coding region of the transcripts (Figure 2A and B). For the mismatched target sites, 4C, 7A, 12U and 16A were picked up, which were distributed evenly across the 19-nt target site and represented as the most disruptive mismatches. Although the mismatches could have affected the activity of the siRNA differently depending on the location of the mismatch and silencing efficacy was compromised gradually with the dilution of the siRNA, elevated mismatch tolerance in the 3′-UTR was still observed for all siRNA concentrations tested (Figure 2A). It showed that mismatches were tolerated at least 3–5 times better in the 3′-UTR than the coding region under the same siRNA concentration (Figure 2B). This indicated that siRNA dosage did not contribute to elevated mismatch tolerance in the 3′-UTR.

### Translational Repression Contributed to Elevated Mismatch Tolerance in the 3′-UTR

**Figure 3:**
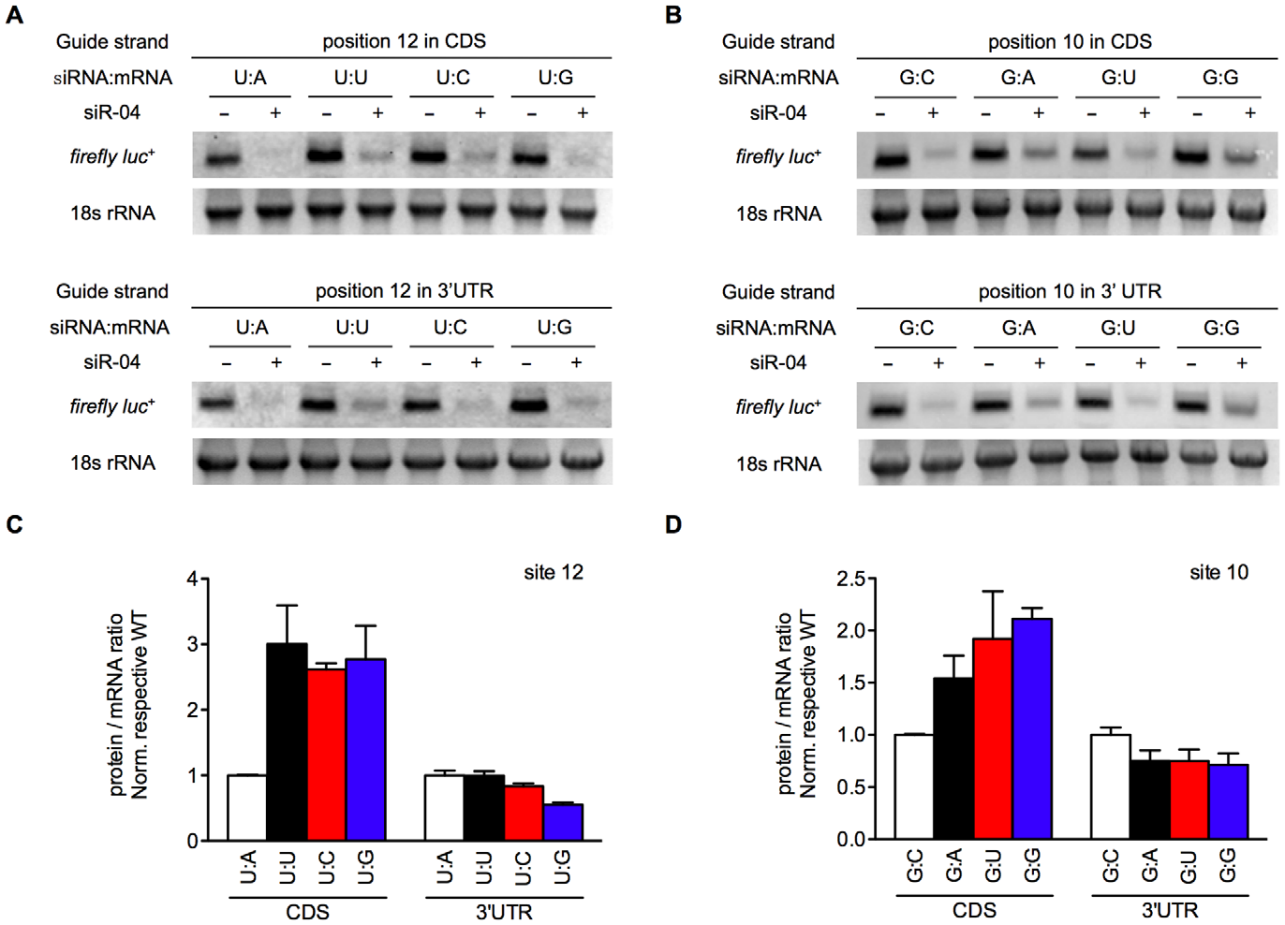
Translational repression contributed to elevated mismatch tolerance in 3′-UTR. (A,B) Northern blot was performed with mRNA transcripts harboring perfectly matched or single-nucleotide mismatched targets of siR-04, located in CDS or 3′-UTR respectively. Reporter plasmids mutated at position 12 or 10 at each location were co-transfected into HEK293A cells with (+) or without (-) the siRNA, and then the amount of reporter mRNA was evaluated using biotin-labeled RNA probe. 18S rRNA was included as loading control. (C,D) Comparison of gene silencing in mRNA and protein levels at position 12 (C) and 10 (D). The intensity of mRNA bands was quantified by ImageJ software, while silencing efficacy of siRNA on protein level was reflected by reporter enzyme activity. The target site location and siRNA:mRNA match pattern were given under the x-axis. The y-axis represented protein yield by the remaining mRNAs, and this repression ratio of siR-04 targeting to mismatched site was normalized to that targeting to perfectly matched site (WT) at either location. Error bars represented SD. All experiments were performed at least twice.

In order to corroborate the results we obtained using reporter enzyme activity as an indicator, we went further to determine the mRNA levels during siRNA-mediated silencing through interaction with different mismatched sites in the coding region and the 3′-UTR. Interestingly, we found that mRNA levels were reduced to identical levels in response to siR-04 knockdown, no matter where the sites were placed and what mismatches were involved (Figure 3A and B). This was clearly different from the protein levels reflected by reporter enzyme activity. These results showed that although siRNA down-regulated mRNA levels to a similar extent when the siRNA target sites were placed in the coding region and the 3′-UTR, the protein levels were disproportionately lower when the siRNA target sites were in the 3′-UTR (Supplementary Figure S3). In other words, the equally remaining mRNAs bearing siR-04 target site with mismatches in the 3′-UTR produced less proteins than that in the coding region (Figure 3C and D). This suggested that the accumulation of translation products of the remaining transcripts were further down-regulated in the 3′-UTR, and the most likely explanation for this is translational repression.

Despite different mismatch tolerance of siR-04 towards the coding region versus the 3′-UTR sites, similar tolerance profile was observed on mismatches in both regions (Supplementary Figure S3). At position 10, target mutation from wild-type C to U (red bars), forming a G:U wobble base-pair between the siRNA guide strand and the target site was shown most tolerated for both coding or 3′-UTR targets; while at position 12, target mutation from wild-type A to G, forming a U:G wobble base-pair was most tolerated (blue bars). This observation corroborated our earlier findings [26] and suggested that the same effector component was involved in gene silencing towards mismatches in both regions.

### AGO2 Contributed Predominately to Silencing Activity in both Regions

**Figure 4:**
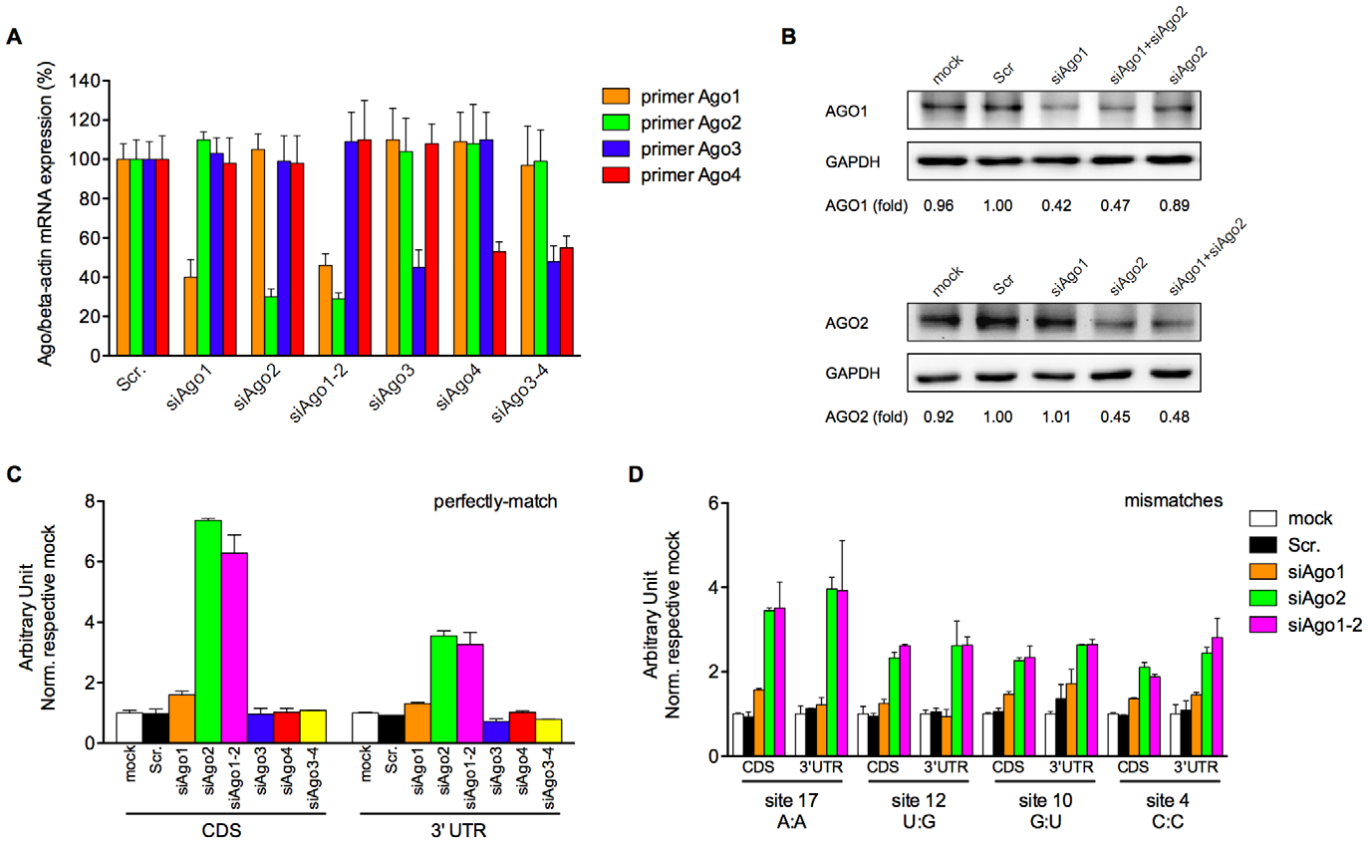
AGO2 contributed predominately to silencing activity in both regions. (A) The relative AGO mRNA levels were measured by quantitative RT-PCR 24 hours post siAgo transfection. Results were average values of assays in triplicates, and all experiments were repeated three times. (B) The relative AGO protein levels were detected by western blot 56 hours post siAgo transfection, at the time point when AGO ablated cells were harvested to evaluate *luciferase* activities. GAPDH was included as loading control. The intensity of protein bands was quantified by ImageJ software (NIH, USA). All experiments were performed at least twice. (C) The normalized silencing efficacies of siR-04 on perfectly matched target in CDS versus 3′-UTR after AGOs ablation. Silencing of AGO expression was carried out by gene-specific siRNA assessed in the previous study [9], and subsequently, influence of the gene silencing on perfectly-match tolerance was evaluated by reporter system. All data were normalized to mock. (D) The normalized silencing efficacies of siR-04 on single-nucleotide mismatched target sites in CDS versus 3′-UTR at the indicated positions (4C, 10U, 12G, 17A) after AGOs ablation. The target site location and siRNA:mRNA match pattern were given under the x-axis. Error bars represented SD. Data were average values of assays in triplicates, and all experiments were repeated at least twice.

In order to obtain mechanistic insight into the elevated mismatch tolerance in the 3′-UTR, especially the potential translational repression in the 3′-UTR by siRNA, we investigated the roles of AGO proteins in the mismatch tolerance of siR-04. Gene-specific siRNAs targeting each AGO protein used in a previous study were taken [9] and the effect of AGO knock-down was assessed at both the mRNA and protein levels (Figure 4A and B). Although the AGO proteins were not completely repressed, due to the limitation of siRNA methodology to knockdown key components of RNAi, this is a practical and rapid strategy to evaluate each AGO function in mismatch tolerance in cultured cells. Gene-specific AGO siRNA, namely siAgo, was transfected into HEK293A cells one day before transfection of siR-04 and its corresponding targets. After AGO ablation, the silencing activity of siR-04 on perfectly-matched targets located in both regions were evaluated. We found that the AGO2 knock-down significantly reduced the activity of siR-04 on perfectly-matched targets located in both regions, resulting in a 7-fold de-repression for the coding region site and a 3.5-fold de-repression for the 3′-UTR site relative to respective mock, the control without siRNA transfection. However, AGO1 had little influence on silencing perfectly-matched site in either region, with only a ∼1.5-fold de-repression after siAgo1 transfection. And AGO3 or AGO4 did not affect siR-04 activity in both regions (Figure 4C).

Then, we investigated the impact of each AGO protein on the mismatch tolerance of siR-04. Four positions along the siRNA target site were chosen, which were distributed in the seed region (position 4), approximating to the cleavage site (position 10 and 12) or 3′-end of the guide strand (position 17), and represented as different kinds of interaction between the siR-04 and the target site. Consistent with the finding for siR-04 towards the perfectly-matched sites, AGO2 contributed predominately to siR-04 activity in the two regions (Figure 4D and Supplementary Figure S4A). The reduction in luciferase activity upon AGO2 ablation relative to respective mock had no significant difference with the position in coding region or 3′-UTR, both a 2-fold de-repression in either location. These results indicated that AGO2 was the major mediator of siRNA-induced silencing at sites located in both coding region and 3′-UTR of a transcript, no matter whether such sites involved a mismatch or not. It was in agreement with our earlier data suggesting that the same effector component contributed to elevated mismatch tolerance in both regions. Similarly, siAgo1 had little impact on siR-04-mediated silencing mismatches in both regions (Figure 4D and Supplementary Figure S4A). In parallel experiments, the impact of knocking down AGO3 and AGO4 was also assessed and it was found that neither AGO3 nor AGO4 alone or in combination had any effect on the silencing of target sites in either region (Supplementary Figure S4B and C).

### siRNA was not Necessarily Acting Better Towards its Perfectly-matched Site in 3′-UTR than in Coding Regions

**Figure 5:**
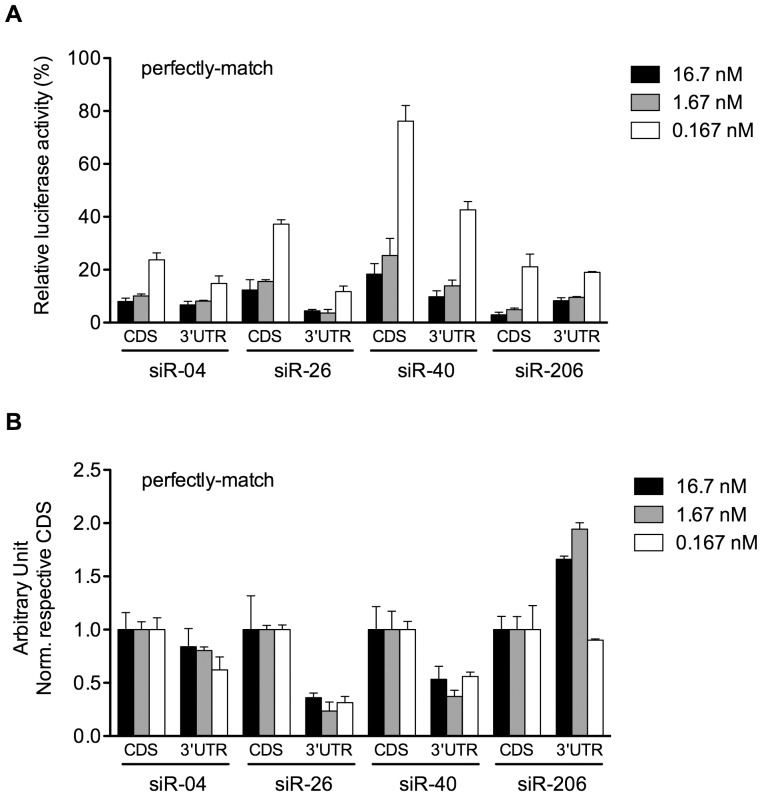
siRNA towards perfectly matched site was not necessarily acting better in 3′-UTR in comparison to CDS. Silencing efficacies of four siRNAs (siR-04, 26, 40, 206) on perfectly matched targets in CDS versus 3′-UTR were shown as (A) original data and (B) normalized data, by using varying siRNA concentrations from 0.167 to 16.7 nM. The normalized data were calculated by normalizing each original silencing efficacy of siRNA towards 3′-UTR site to that towards CDS site. The lower bars indicated siRNA had stronger silencing activities. Error bars represented SD. Data were average values of assays in triplicates, and all experiments were repeated at least twice.

Through sufficient comparison of mismatch tolerance properties of siRNA on targeting sites in CDS versus 3′-UTR, it was found that the silencing activity of siRNA on mismatched sites was universally much higher in 3′-UTR than that in coding region. However, how about the silencing efficiency of siRNA towards perfectly-matched site in either region? Therefore, we compared the silencing efficacy of four siRNAs, siR-04, siR-26 and siR-40, as well as siR-206 on perfectly-matched sites located in both regions in the 100-fold dilution ranges of the siRNA. It showed that siR-04 had very tiny different activities on perfectly-matched site located in the coding region versus 3′-UTR, with only slightly better potency towards site in the 3′-UTR. However, siR-206 showed a reverse response on silencing its perfectly-matched target compared to siR-04. The silencing efficiency of siR-206 towards the coding region site was 1.5 times better than that towards the 3′-UTR site at the siRNA final concentration of 16.7 nM and 1.67 nM. siR-26 and siR-40 were more efficient when targeting 3′-UTR sites over two orders of magnitude of siRNA concentration (Figure 5). This discrepancy may derive from the intrinsic activity of siRNAs. These results demonstrated that not all of siRNAs acted more efficiently towards perfectly-matched sites in the 3′-UTR.

## Discussion

Our previous studies on siRNA specificity have established a practical guideline for allele-specific siRNA design. Recently, some SNPs located in the 3′-UTR of genes were found to affect mRNA stability or be associated with susceptibility to diseases [31]–[33] However, there is no evidence that allele-specific siRNAs can also efficiently discriminate SNPs in 3′-UTR. The current paper analyzed the silencing properties of a number of siRNAs when they were targeting sites located in the 3′-UTR in comparison to their silencing properties when targeting coding region sites in order to establish knowledgebase for how siRNA behave in the 3′-UTR region in term of specificity and potency.

By systematic comparison of mismatch tolerance properties of siRNA on targeting sites in the coding region versus 3′-UTR, it was demonstrated that siRNAs suffered universally from astonishing losses of specificity in terms of discrimination of single nucleotide mismatches. We went further to explore the mechanism governing the silencing efficacy on these sites. Our data demonstrated that for all mismatched sites, the silencing of AGO2 had the greatest impact on the silencing activity, which is consistent with previous reports [34], [35]. But surprisingly AGO2 ablation only caused a moderate decrease in the absolute silencing activity of siRNA on matched and mismatched sites placed in the 3′-UTR. In contrast, similar levels of AGO2 knock-down greatly diminished the RNAi effects of the same siRNA on the same target site located in the coding region (Supplementary Figure S4A). We compared the mRNA levels and reporter enzyme levels, and obtained evidence that the actual mRNA levels after silencing were virtually the same for mismatched sites located in both regions, although significantly higher silencing efficacy was found for the reporter enzyme levels, which reflects the actual protein levels when the target sites are located in the 3′-UTR. This strongly suggested that the difference in siRNA silencing of mismatched sites located in the coding region or 3′-UTR was not due to different slicer activity, but due to translational repression activity that only occurred in the 3′-UTR, in a way similar to miRNA, which represses translation primarily in the 3′-UTR [19], [30], [36]. In summary, the current work revealed that siRNAs appear to have elevated mismatch tolerance when targeting 3′-UTR sites, most likely due to translational repression in addition to mRNA degradation. While such a phenomenon gave us a hint that 3′-UTR may not be suitable for designing allele-specific siRNA to discriminate SNPs from wild-type alleles, it might worth testing whether such a trend can facilitate the design of siRNA that would target multiple genes in a large gene family.

## Materials and Methods

### Oligonucleotides

DNA oligonucleotides were obtained from Invitrogen (Beijing, China); RNA oligonucleotides were from RiboBio (Guangzhou, China) and Genepharma (Shanghai, China). The sequences of siRNAs and their matched targets, as well as human AGO1–4 specific siRNAs were included in Supplementary Table S1.

### Construction of siQuant Vector

A previously reported siRNA validation vector siQuant [37] was used in the study to assess the gene silencing efficacy of synthetic siRNA in coding region. The wild-type targets, as well as single-nucleotide mutated targets were cloned in-frame to form fusion reporter gene with target site in coding region.

To assess the silencing activity in 3′-UTR, modifications were made in siQuant vector. For cloning target sequence in different positions of 3′-UTR, a *Bgl*II-*Apa*I cloning site was inserted downstream of the stop codon, and the original cloning site was destroyed. Furthermore, an irrelevant fragment of 1299 bp was amplified from the 3′-UTR of mouse *Esrrb* gene (NM_011934) and inserted upstream of the cloning site to space it from the stop codon. The same sequence was also inserted downstream of the cloning site to space it from poly(A) signal. Perfectly matched or single-nucleotide mutated target sequences were accordingly inserted into the cloning site, forming various fusion reporter genes.

### RNA Interference Assay

Human embryonic kidney cells (HEK293A) were grown in Dulbecco’s modified Eagle’s medium (HyClone®, Thermo, Beijing, China) supplemented with 10% fetal bovine serum (Sigma-Aldrich, Saint Louis, MO), 100 units/ml penicillin and 100 µg/ml streptomycin (Gibco, Invitrogen, Grand Island, NY). The cells were seeded into 24-well plates at a density of ∼1×10^5^ cells/well one day before transfection. Reporter plasmid (100 ng/well) carrying siRNA target was transfected into the cells at approximately 50% confluence using Lipofectamine™ 2000 (1 µl/well, Invitrogen, Carlsbad, CA), together with pRL-TK control vector (50 ng/well), with or without the siRNA (16.7 nM).

Twenty-four hours after transfection, the activities of both luciferases were determined by a fluorometer (Synergy™ HT, BioTek, Winooski, VT). The *firefly* luciferase signal was normalized to the *renilla* luciferase signal for each individual well, and the silencing efficacy of each siRNA was calculated by comparison with a sample without siRNA treatment. All experiments were performed in triplicates and repeated at least twice.

### Northern Blot

Twenty-four hours after transfection of reporter plasmid and siRNA, total RNA was harvested from HEK293A cells with TRIzol® Reagent (Invitrogen, Carlsbad, CA) according to the manufacturer’s instructions. Total RNA (10–15 µg) was separated by electrophoresis on a 1% denaturing formaldehyde/MOPS agarose gel containing ethidium bromide then transferred onto a charged nylon filter (Millipore, Billerica, MA). The intensities of 18S and 28S rRNA bands were checked under the ultraviolet light to verify that all samples were loaded equally and that no RNA degradation had occurred. The Biotin-16-UTP (Roche, Quebec, Canada) labeled RNA probe (763 bp, covered nucleotides 406–1168 on basis of Genbank accession number U47296) was generated by the Riboprobe® *in vitro* transcription system (Promega, Madison, WI). Hybridization and stringent washing were performed according to DIG easy Hyb (Roche, Indianapolis, IN), and the signals were detected by Streptavidin-IRDye 800 CW (LI-COR, Lincoln, NE) on an Odyssey® Infrared Imaging System (LI-COR, Lincoln, NE). All experiments were performed at least twice.

### AGO Ablation

For the ablation of Argonaute proteins, AGO-specific siRNA (100 nM) was transfected into HEK293A cells, which were seeded into 6-well plates one day before, using Lipofectamine™ 2000 (4 µl/well, Invitrogen). Twenty-four hours after siAgo treatment, one portion of cells were lysed to detect mRNA levels of AGOs by RT-PCR, the others were split and seeded into 6-well plates or 24-well plates at a density of 5×10^5^ cells/well or 1.2×10^5^ cells/well, respectively. Cells in 6-well plates were cultured another 24 hours to detect protein levels of AGOs by western blot, while cells in 24-well plates were allowed to adhere to the plates for 6 hours then treated with reporter plasmid carrying siRNA target site, pRL-TK control vector, with or without siRNA (16.7 nM) to perform dual-luciferase assay on the following day.

### Quantitative RT-PCR

Twenty-four hours after transfection of 100 nM AGO-specific siRNAs, total RNA was harvested from HEK293A cells with TRIzol® Reagent (Invitrogen) and converted into cDNAs by ImProm-II™ Reverse Transcription System (Promega, Madison, WI). Real time PCR was then performed using SYBR® Green I dye (Invitrogen, Carlsbad, CA) to detect the mRNA expression levels of human AGOs and β-actin, an internal control. The results were normalized to that transfected by scrambled control siRNA. All experiments were performed in triplicates and repeated at least triples. Primer sets used in the detection assessed in the previous report [9] are included in Supplementary Table S1.

### Western Blot

Fifty-six hours after transfection of 100 nM AGO-specific siRNAs, total proteins were extracted from HEK293A cells by cell lysis buffer (Cell Signaling, Danvers, MA). Isolated proteins (20–40 µg) were separated by electrophoresis on a 10% SDS polyacrylamide gel and transferred onto a nitrocellulose filter membrane (Millipore, Billerica, MA). The membrane was blocked and incubated at 4°C overnight with an antibody reacting with human AGO1 (mouse anti-hAgo1, Cat.07-599, Upstate, Millipore, Temecula, CA), human AGO2 (mouse anti-hAgo2, Cat.H00027161-M01, Abnova, Taipei, Taiwan), or GAPDH (mouse anti-hGAPDH, Cat.sc-137179, Santa Cruz, Santa Cruz, CA). Then the membrane was washed and incubated at room temperature with secondary antibody conjugated with horseradish peroxidase (goat anti-mouse IgG1-HRP, Cat.sc-2060, Santa Cruz, Santa Cruz, CA). The signals were detected by ECL kit (Cat.sc-2048, Santa Cruz, Santa Cruz, CA) on an imaging system (Universal Hood II, Bio-Rad, Segrate, Italy), and the intensity of protein bands was quantified by ImageJ software (NIH, USA). All experiments were performed at least twice.

## Supporting Information

Figure S1 **The secondary structures of the transcripts bearing siRNA target sites in different locations.** (A) siR-04 target site in CDS; (B) siR-04 target site in 3′-UTR; (C) siR-04 target site far from the stop codon within the 3′-UTR; (D) siR-04 target site far from the poly(A) signal within the 3′-UTR. The secondary structures of these mRNAs were predicted by software of RNAstructure 5.3 [38]. The blue lines indicated siR-04 and the solid red dot indicated 5′-end of the siRNA antisense strand.
(TIF)

Figure S2 **Positional effects of elevated mismatch tolerance in 3′-UTR was a universal phenomenon.** (A–H) The original silencing efficacies of siRNAs on single-nucleotide mismatched targets in CDS (left) or 3′-UTR (right). (A) siR-04 on CDS sites; (B) siR-04 on 3′-UTR sites; (C) siR-26 on CDS sites; (D) siR-26 on 3′-UTR sites; (E) siR-40 on CDS sites; (F) siR-40 on 3′-UTR sites; (G) siR-206 on CDS sites; (H) siR-206 on 3′-UTR sites. The figure was plotted against the position (numbered from the start of the siRNA antisense strand) and the identity of the mismatched nucleotides. The position and the wild-type target sequence were given under the x-axis. The y-axis represented the remained luciferase activity, a ratio of *firefly* luciferase signal/*renilla* luciferase signal. The lower of the bars indicated siRNA had stronger silencing activities. White bars indicate wild-type targets; colored bars indicate single-nucleotide mismatched targets. Error bars represented SD. Data were average values of assays in triplicates, and all experiments were repeated at least twice.
(TIF)

Figure S3 **Comparison of gene silencing in mRNA and protein levels at position 12 (A) and 10 (B).** The intensity of mRNA bands was quantified by ImageJ software, while silencing efficacy of siR-04 on protein level was reflected by reporter enzyme activity. The target site location and siRNA:mRNA match pattern were given under the x-axis. The y-axis represented the remaining reporter gene expression in protein and mRNA levels, respectively. Error bars represented SD. All experiments were performed at least twice.
(TIF)

Figure S4 **AGO2 contributed predominately to silencing activity in both regions, while down-regulated AGO1/3/4 had little impact.** (A) The original silencing efficacies of siR-04 on perfectly matched or single-nucleotide mismatched target in CDS versus 3′-UTR after AGOs ablation. (B,C) AGO3 and AGO4 do not contribute to the translational repression on single-nucleotide mismatched targets. Silencing of AGO3 and AGO4 expression was carried out by gene-specific siRNAs, and subsequently, influences of the gene silencing were evaluated on single-nucleotide mismatched targets at position 4 (B) and 10 (C). All data were normalized to mock. Error bars represented mean SD. Data were average values of assays in triplicates, and all experiments were repeated at least twice.
(TIF)

Table S1 Sequences of siRNAs, miRNAs and DNA oligos used in this study. The underlined sequences represented the interaction region between siRNA and its target site, and the two terminals of target oligos are cohesive ends of *Bgl*II and *Apa*I (labeled in bold).
(DOC)
