# Supplementary figures and images for: siRNA Has Greatly Elevated Mismatch Tolerance at 3′-UTR Sites
Source: PLoS One. 2012 Nov 8;7(11):e49309. doi: 10.1371/journal.pone.0049309 (PMC3493533; doi:10.1371/journal.pone.0049309)

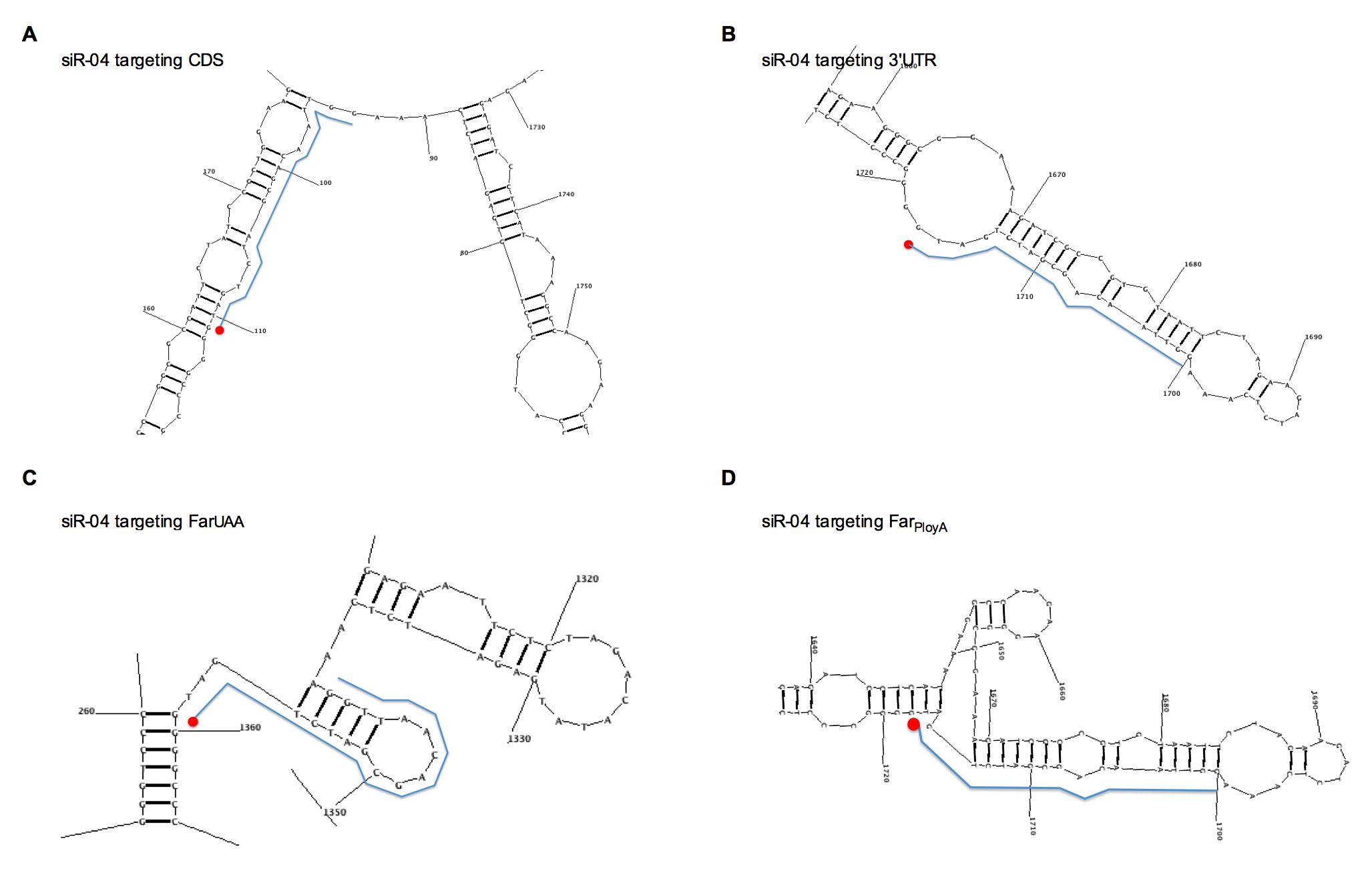

Supplement: Figure S1 — The secondary structures of the transcripts bearing siRNA target sites in different locations. (A) siR-04 target site in CDS; (B) siR-04 target site in 3′-UTR; (C) siR-04 target site far from the stop codon within the 3′-UTR; (D) siR-04 target site far from the poly(A) signal within the 3′-UTR. The secondary structures of these mRNAs were predicted by software of RNAstructure 5.3 [38]. The blue lines indicated siR-04 and the solid red dot indicated 5′-end of the siRNA antisense strand. (TIF) [file pone.0049309.s001.tif]

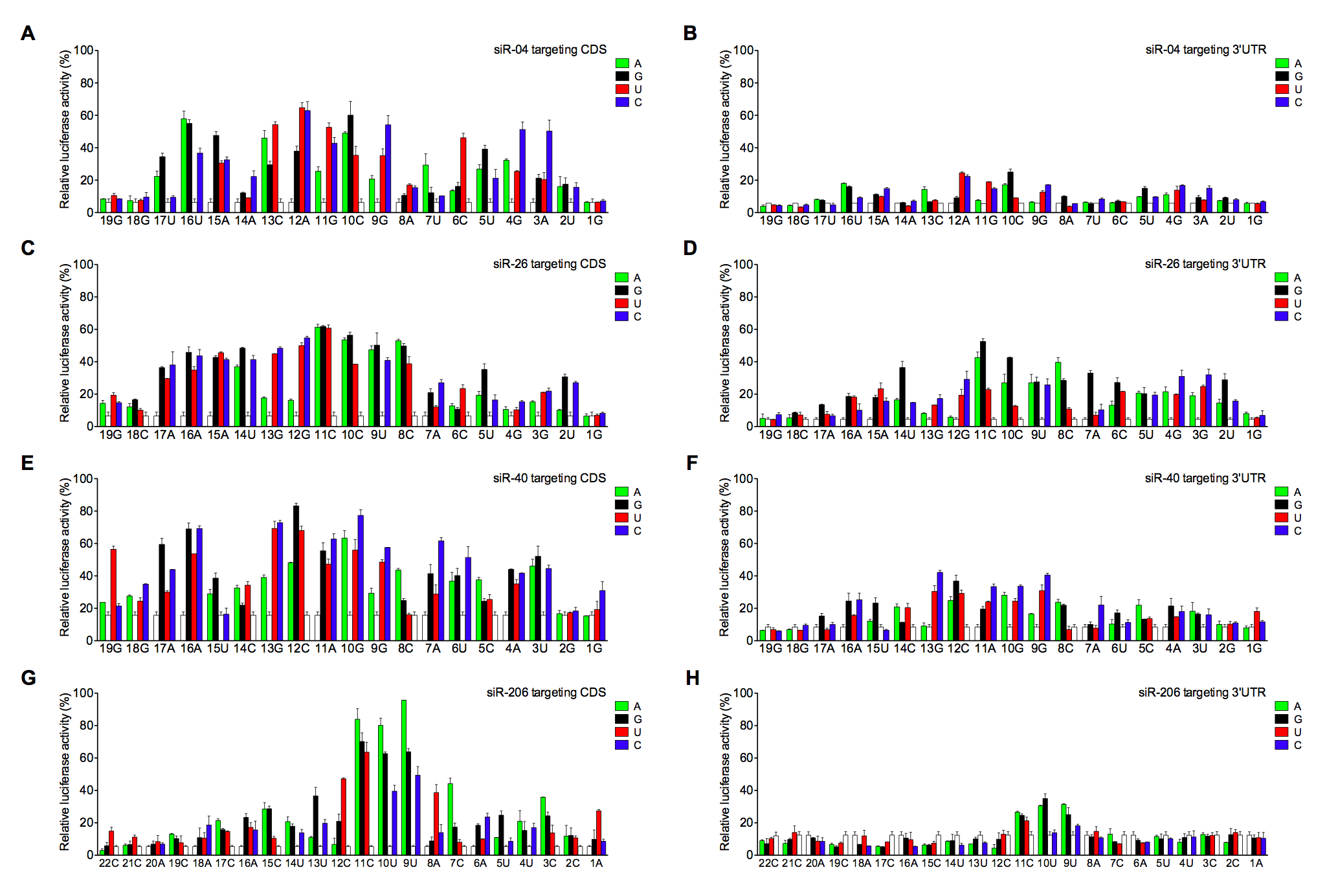

Supplement: Figure S2 — Positional effects of elevated mismatch tolerance in 3′-UTR was a universal phenomenon. (A–H) The original silencing efficacies of siRNAs on single-nucleotide mismatched targets in CDS (left) or 3′-UTR (right). (A) siR-04 on CDS sites; (B) siR-04 on 3′-UTR sites; (C) siR-26 on CDS sites; (D) siR-26 on 3′-UTR sites; (E) siR-40 on CDS sites; (F) siR-40 on 3′-UTR sites; (G) siR-206 on CDS sites; (H) siR-206 on 3′-UTR sites. The figure was plotted against the position (numbered from the start of the siRNA antisense strand) and the identity of the mismatched nucleotides. The position and the wild-type target sequence were given under the x-axis. The y-axis represented the remained luciferase activity, a ratio of firefly luciferase signal/renilla luciferase signal. The lower of the bars indicated siRNA had stronger silencing activities. White bars indicate wild-type targets; colored bars indicate single-nucleotide mismatched targets. Error bars represented SD. Data were average values of assays in triplicates, and all experiments were repeated at least twice. (TIF) [file pone.0049309.s002.tif]

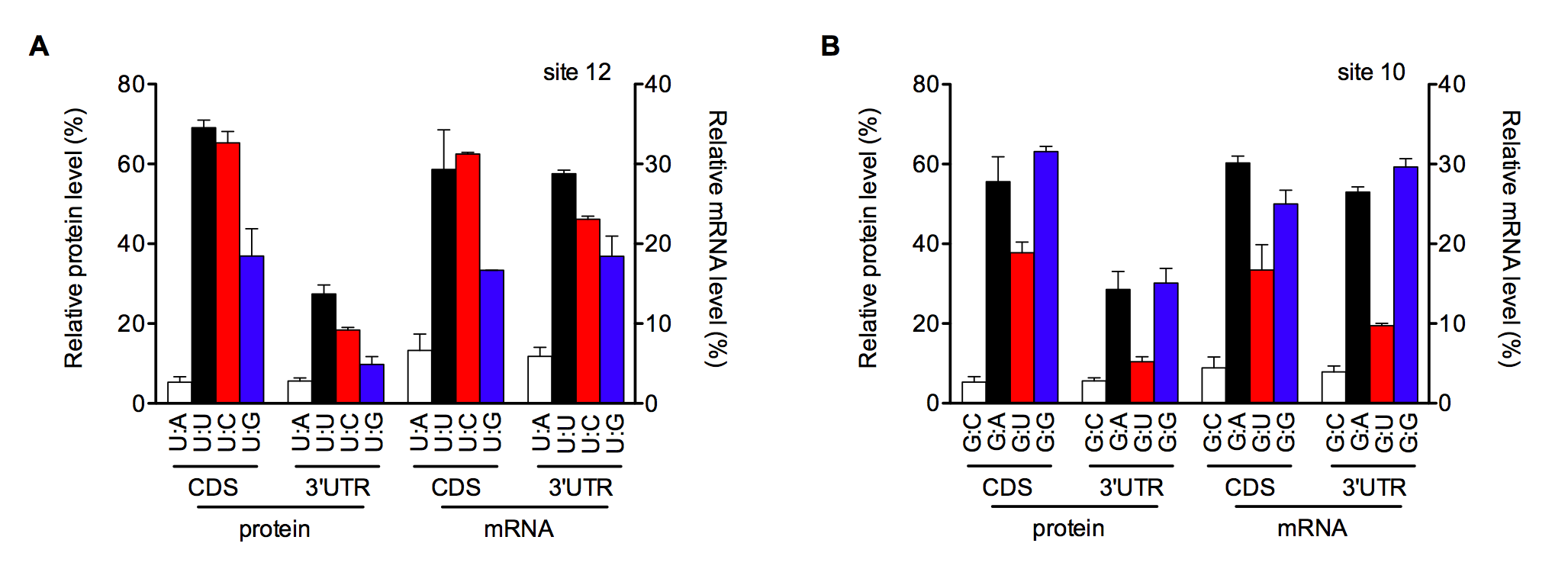

Supplement: Figure S3 — Comparison of gene silencing in mRNA and protein levels at position 12 (A) and 10 (B). The intensity of mRNA bands was quantified by ImageJ software, while silencing efficacy of siR-04 on protein level was reflected by reporter enzyme activity. The target site location and siRNA:mRNA match pattern were given under the x-axis. The y-axis represented the remaining reporter gene expression in protein and mRNA levels, respectively. Error bars represented SD. All experiments were performed at least twice. (TIF) [file pone.0049309.s003.tif]

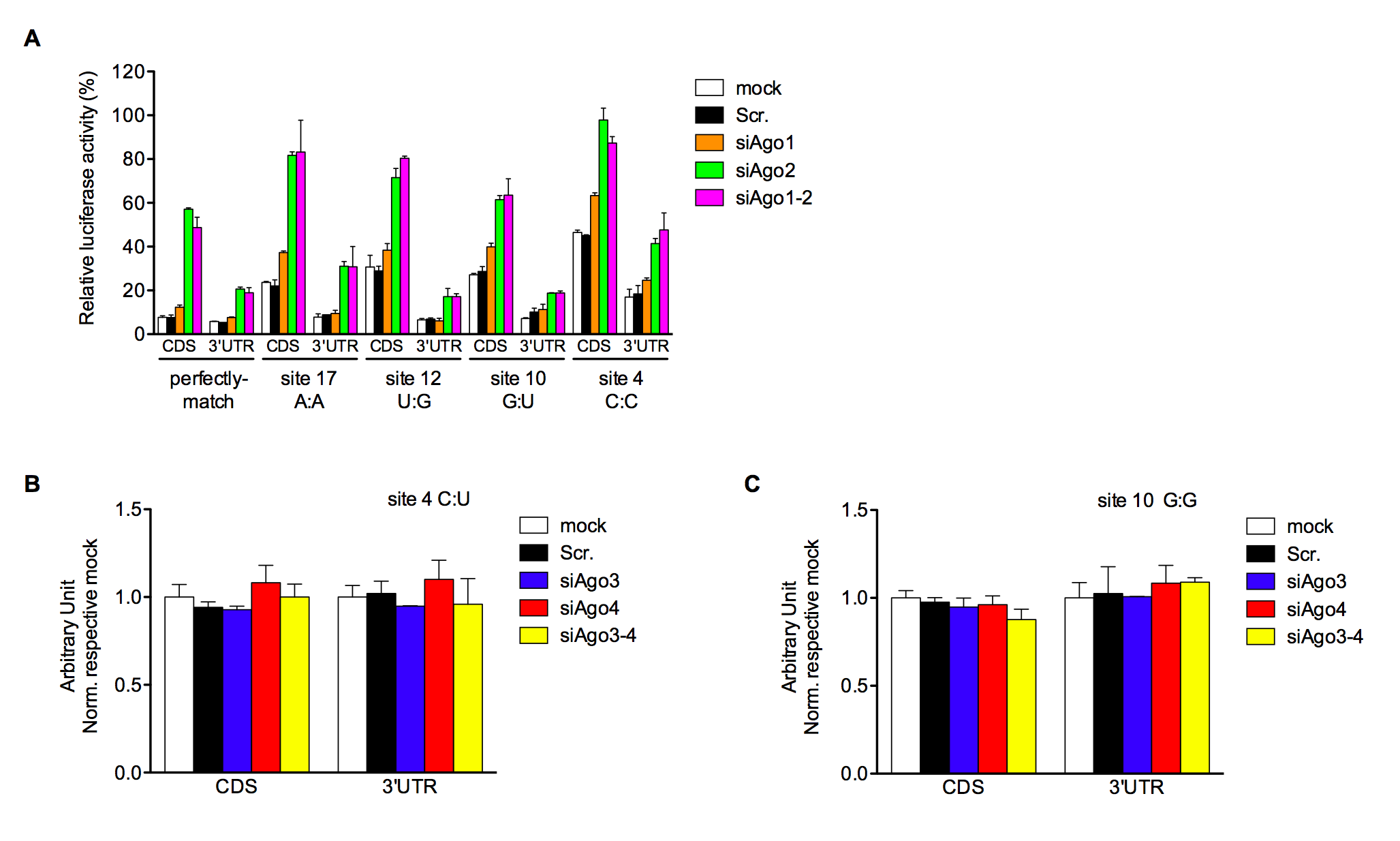

Supplement: Figure S4 — AGO2 contributed predominately to silencing activity in both regions, while down-regulated AGO1/3/4 had little impact. (A) The original silencing efficacies of siR-04 on perfectly matched or single-nucleotide mismatched target in CDS versus 3′-UTR after AGOs ablation. (B,C) AGO3 and AGO4 do not contribute to the translational repression on single-nucleotide mismatched targets. Silencing of AGO3 and AGO4 expression was carried out by gene-specific siRNAs, and subsequently, influences of the gene silencing were evaluated on single-nucleotide mismatched targets at position 4 (B) and 10 (C). All data were normalized to mock. Error bars represented mean SD. Data were average values of assays in triplicates, and all experiments were repeated at least twice. (TIF) [file pone.0049309.s004.tif]
