# Supplementary material for: siRNA Has Greatly Elevated Mismatch Tolerance at 3′-UTR Sites
Source: PLoS One. 2012 Nov 8;7(11):e49309. doi: 10.1371/journal.pone.0049309 (PMC3493533; doi:10.1371/journal.pone.0049309)
Supplement: Table S1 — Sequences of siRNAs, miRNAs and DNA oligos used in this study. The underlined sequences represented the interaction region between siRNA and its target site, and the two terminals of target oligos are cohesive ends of BglII and ApaI (labeled in bold). (DOC) [file pone.0049309.s005.doc]

**Supplementary Table S1. Sequences of siRNAs and DNA oligos used in this study. The underlined sequences represented the interaction region between siRNA and its target site, and the two terminals of target oligos are cohesive ends of *Bgl*II and *Apa*I (labeled in bold).**

| **siRNA guide strand** | **sequence (5’---3’)** |
| --- | --- |
| siR-04 | 5’-CAUCAGAUCGCUGUUAACCdtdt |
| siR-26 | 5’-CACCAGUGAGGCCAUUUGCdtdt |
| siR-40 | 5’-CCAUGAUGCCUGCGAUUCCdtdt |
| siR-206 | 5’-UGGAAUGUAAGGAAGUGUGUGG |
| scrambled siRNA | 5’-ACGUGACACGUUCGGAGAAdtdt |
| siAgo1 | 5’-UUCUUGAGCACCUCUUCUCdtdt |
| siAgo2 | 5’-UUCAGAUGGACUUCCGUGCdtdt |
| siAgo3 | 5’-UUGUGCGUAAGGUAUCUUGdtdt |
| siAgo4 | 5’-AUUGCUAUUAGUUCUGGCCdtdt |
|  |  |
| **siRNA target**  **(sense strand)** | **sequence (5’---3’)** |
| target of siR-04 | 5’-**GATCT**CAAAGGTTAACAGCGATCTGATG**GGGCC** |
| target of siR-26 | 5’-**GATCT**CAGCAAATGGCCTCACTGGTGCC**GGGCC** |
| target of siR-40 | 5’-**GATCT**CAGGAATCGCAGGCATCATGGCC**GGGCC** |
| target of siR-206 | 5’-**GATCT**CACCACACACTTCCTTACATTCCACC**GGGCC** |
|  |  |
| **real-time PCR primers** | **sequence (5’---3’)** |
| RT-Primer-Ago1-F | 5’-GCACTGCCCATTGGCAACGAA |
| RT-Primer-Ago1-R | 5’-CATTCGCCAGCTCACAATGGCT |
| RT-Primer-Ago2-F | 5’-CGCGTCCGAAGGCTGCTCTA |
| RT-Primer-Ago2-R | 5’-TGGCTGTGCCTTGTAAAACGCT |
| RT-Primer-Ago3-F | 5’-GGAATTAGACAAGCCAATCAGCA |
| RT-Primer-Ago3-R | 5’-AGGGTGGTCATATCCTTCTGGA |
| RT-Primer-Ago4-F | 5’-CTAACAGACTCCCAGCGTGTCA |
| RT-Primer-Ago4-R | 5’-GACTGGCTGGCCGTCTAGTCA |
| RT-Primer-β-actin-F | 5’-CCAACCGCGAGAAGATGA |
| RT-Primer-β-actin-R | 5’-CCAGAGGCGTACAGGGATAG |
|  |  |
| **PCR primers** | **sequence (5’---3’)** |
| FF *luc*^+^-probe-F | 5’-ACAGATGCACATATCGAGGTGGA |
| FF *luc*^+^-probe-R | 5’-TGGCGAAGAAGGAGAATAGGGTT |
| mouse Esrrb-PstI-F | 5’-GACCCTGCAGTCTGGTTGTC |
| mouse Esrrb-EcoRI-R | 5’-CTCTGAATTCTCCTGTCTCCTTGT |
